# Incorporating patient and family preferences into evidence-based medicine

**DOI:** 10.1186/1472-6947-13-S3-S6

**Published:** 2013-12-06

**Authors:** Laura A Siminoff

**Affiliations:** 1 Department of Social and Behavioral Health, School of Medicine, Virginia Commonwealth University, Richmond, VA – 23219, USA

## Abstract

**Background:**

Clinicians are encouraged to practice evidence-based medicine (EBM) as well as patient-centered medicine. At times, these paradigms seem to be mutually exclusive and difficult to reconcile. It can become even more challenging when trying to include the preferences of the patient’s family members. This paper discusses the basis for this quandary, providing examples of the real-world impact it has on diagnosis-seeking and treatment decision-making behaviors and how it might inform implementation of EBM practices.

**Analysis:**

To further explore the role of friends and family in health-care decision making and to understand how patients and families introduce other considerations that may or may not be congruent with a strictly EBM approach, data from two research studies that examined healthcare–seeking behaviors are presented. Both studies explore how family and friends not only can influence health-care decisions but also may be a source of conflict for the patient and/or clinician.

**Conclusions:**

Illness is a biological and social process. Clinicians who engage in EBM need to acknowledge the social and cultural factors that affect the health-care encounter, understand the important role of those factors in health-care decision making, and expand the paradigm of EBM to incorporate sociocultural influences more explicitly. Moreover, recognition of the influences family members and other caregivers have within the clinical encounter—by offering opinions and participating in treatment-related decision making—is needed and could lead to more efficient and effective health care.

## Background

Evidence-based medicine (EBM) has been defined as combining the best evidence available from systematic research with clinician expertise to treat patients; consequently, Sackett et al. contend that both are essential [1]. Without incorporating the most current, up-to-date evidence, health care quickly becomes outdated. Conversely, without clinical expertise, clinicians may be driven by research evidence when it is not appropriate for or applicable to an individual patient [1]. This view of EBM, however, is limited and confines it to a purely biomedical approach that does not readily or explicitly incorporate the patient’s perspective. When the clinician does not actively engage the patient, EBM can effectively diminish treatment decisions to just the “evidence.” To prevent a new reductionist approach to medical practice, clinical expertise and the explicit addition of patient preferences and values are needed to temper how the evidence is applied to the individual patient. Moreover, in many clinical contexts, evidence may be sparse or unavailable. Under these circumstances, the patient’s perspective is critical if we are to avoid paternalism.

Patient-centered care, by contrast, invites patients to be active participants in their care [2]. Patient-centered care emphasizes the patient’s experience with their illness [3]. As such, clinicians practicing patient-centered care consider the biopsychosocial aspects of the illness, and treatment decisions are made with the patient and with consideration of the patient’s preferences and values [4]. Research suggests that patient-centered care increases treatment adherence and leads to better outcomes [4-8]. However, some argue that patient-centered care lacks evidence and is a “fuzzy concept” [9]. At its most extreme, clinicians could be viewed as merely “advisors” or technicians delivering a service. However, the ideal is to bring patients into the process—to the extent desired by the patient—and to use the expertise of both interactants. One of the most important aspects of patient-centered care is the facilitation of shared decision making.

At either extreme, it is important to recognize that physicians and patients may approach the clinical encounter with different priorities. Physicians often seek to diagnose and treat an illness based on the patient’s symptoms and objective information derived from physical examinations, laboratory tests, or the patient’s medical history. Conversely, patients may only seek care when symptoms signal there is a problem because of disruption of their work or social life or when others notice a problem. Often, this information-seeking behavior of patients is driven by a desire to understand and “make sense” of their condition. These self-explanations or the beliefs a patient holds about his/her condition have been described by Kleinman et al. as the Patient Explanatory Model of Illness [10]. Explanatory models can significantly influence the clinical encounter, as well as a patient’s overall health behavior. For example, if a patient believes her illness or symptoms are signs of “female trouble,” she may be inclined to only share information with the clinician as it supports this assumption or explanation. Another example is a patient with cancer who assumes pain is a “normal” part of the cancer experience. This belief may lead the patient to decide not to explore the pain with the physician. Explanatory models, therefore, tend to be rooted in lived sociocultural experiences, as influenced by friends and family.

When physicians and patients have different expectations or hold dissimilar beliefs about the diagnosis and treatment of an illness, the result can be dissonance and conflict. For example, Loewe and Freeman reported that whereas patients with diabetes are concerned about preventing limb loss, physicians are more concerned about protecting internal organs from damage [11]. This dynamic can become more complex as physicians attempt to practice EBM, especially if their priorities fail to align with those of their patients. This misalignment may lead to stress and tension between physicians and patients, resulting in situations where patients may not follow the treatments recommended by their physicians and often do so without their physicians’ knowledge. It can also result in dysfunctional behaviors, such as doctor shopping or avoidance of the health-care system.

Is it possible for these two *seemingly* opposing paradigms—EBM and patient-centered care—to be integrated? As Bensing suggested, EBM can become more patient-centered by considering and incorporating patient perspectives into clinical trial designs and by allowing patients with strong preferences to select their treatment group while those without strong preferences are randomized [9]. While patient-preference study designs do not replace randomized controlled trials, they can complement them and provide greater insight into patient choices and their potential interaction with outcomes [12]. Similarly, patient-centered care can become more evidence based by using rigorous communication study designs and by incorporating communication research into health services research [9]. This proposition is not to say that these paradigms never align, because in some cases they do. In other cases, however, the paradigms appear to be at odds, and clinicians are challenged to “bridge the gap.” We contend that the goal of patient-centered EBM can be achieved through the incorporation of patient-derived data. This article discusses the data needed to expand the EBM paradigm, including the role of patients’ families and their contributions to diagnosis seeking and treatment-related decision making.

## Influence and role of the family

The influence of the family in making health-care decisions and requests has not received the attention it deserves. It has been estimated that 50% of healthy outpatients, 65 years of age and older, have some family involvement in their medical care [13]. When patients are very ill, such as with cancer, family involvement frequently increases [14]. Family members can play many roles, and these roles may be determined by a number of factors, including the patient’s disease or the type of health-care decision under consideration (e.g., screening, acute or chronic illness). Lewis et al. found that more than a third of cancer patients (33.9%) cite family members, friends, and coworkers as a primary source of health information [15]. However, the literature has not adequately addressed the impact family members may have on health and medical decision making.

To further explore the role of friends and family and to understand how patients and families introduce other considerations that may or may not be congruent with a strictly EBM approach, data from two research studies that examined health-care–seeking behaviors are analyzed. They serve to illustrate how family and friends may act as sources of information and advice—either confirming or disconfirming patients’ explanatory models of illness and in turn can influence health-care decisions. The first study explores how patients newly diagnosed with colorectal cancer (CRC) assessed and sought treatment for the disease. The second study explored communication congruence about treatment between patients with late-stage lung cancer and their family caregivers. Both studies examined how family and friends (sometimes referred to as “confidants”) can not only influence medical decisions (i.e., diagnosis seeking or treatment) in ways that may or may not align with EBM recommendations or guidelines but also can serve as a source of conflict.

## Methods

### Study 1: Diagnosis-seeking behavior in patients with colorectal cancer

The study methods have been reported in detail elsewhere [16]. In brief, semi-structured interviews, approximately 2 hours in length, were conducted with 242 patients who had been diagnosed with stage 1, 2, 3, or 4 colon cancer within the prior 6 months. The interviews were conducted, on average, 4 months after diagnosis.

Study participants were recruited at five sites across Virginia and Ohio. Potential participants were identified and recruited based on a review of medical records from the sites. Patients were screened via telephone to determine their eligibility and interest in study participation. Interviews focused on patient experiences in recognizing symptoms and actions related to symptom appraisal. Because the study was exploring the patient’s role in diagnosis, patients diagnosed with CRC through routine screening were excluded from the study. Data were also abstracted from the medical records of every provider each patient consulted with regarding symptoms.

Of the patients interviewed, 52.1% were male and 53.3% were married. Over half of the patients (52.1%) had more than a high school education; 32.4% had completed some college courses, 10.3% had a bachelor’s degree, and 9.1% had completed postgraduate studies. Many were employed (45.5%) and had private health insurance (45%). The average age of the patients was 58 years, with 43% self-identifying as African American and 53% self-identifying as white. Most patients had stage 2 (24.8%) or stage 3 (39.9%) CRC at the time of diagnosis [16].

### Study 2: Treatment-related decision making in patients with lung cancer

Patients with lung cancer (n = 184) and a matched set of caregivers were recruited from three sites in the Cleveland, Ohio, metropolitan area. Patients were identified by reviewing medical charts at the three sites and, after obtaining physician permission, were contacted by mail with information about the study. Patients were then telephoned to provide additional study details and to obtain consent. Patients consenting to participate in the study were asked to identify their primary caregiver, who was also contacted to obtain his/her consent. Semi-structured interviews with the caregiver and the patient were conducted separately using an instrument we developed to capture family, caregiver, and patient communication conflicts. Interviews focused on treatment-related decisions, including routine treatment decisions such as: where, when and how treatment will be administered; weighing the benefits and side effects of treatment; and decisions about entering hospice care. A detailed description of the methods and procedures are published elsewhere [17].

Most patients interviewed were male (54%), while most caregivers were female (75%). The average age of the patients was 65 years, while the average age of the caregivers was 56 years. The proportion of African Americans in the patient population was higher than in the caregiver population (24% vs. 12%). More than half of the caregivers (58%) were the patients’ spouse, while the remaining caregivers were identified as a child, parent, or significant other.

The relevant institutional review boards approved both studies, and all participants provided written informed consent.

## Results

### Seeking a diagnosis

People use social cues in addition to bodily cues to decide whether or not to explore symptoms within the formal health-care system. This process is referred to as “symptom appraisal.” Symptom appraisal is affected by the opinions, advice, and preferences of an individual patient’s confidants and the explanatory model the patient holds. For example, the explanatory model can direct an individual to view his/her symptoms under several rubrics. In our sample of CRC patients, the rubrics were: acute illness; a chronic illness or health problems that are caused by lifestyle or somehow “run in families”; or health issues that were specific to women. Examples of how each rubric was constructed are provided in Table 1.

**Table 1:** Colorectal cancer explanatory models

Explanatory models	Examples
Acute illness	Patient: “So I’m thinking ‘It’s probably a bug. It’s probably going to work itself out on its own, and if it doesn’t, I’m going to go to the doctor…’”
Chronic illness	Patient: “I had a neighbor like several of my neighbors when I mentioned it said that literally everyone on the block…someone in their family had IBS. I thought that’s what it was.”
“Female” problems	Patient: “They just knew I wasn’t feeling good, and I told them I was having cramps. You know, sometimes those cramps just felt almost like labor paints. That’s why I thought it could be that cyst [that] was on my ovary.”

When a set of symptoms is recognized by a patient, counsel can be sought from several sources, including family, friends, or coworkers (confidants) or through secondary sources such as the Internet. Among the patients in the CRC study, 25% sought information this way [18]. Most frequently, the patients with CRC sought guidance from confidants. Advice was generally based on the experiences of the confidant or others with the symptoms the patient described and was generally provided as casual advice. In some instances, especially when closer confidants such as a spouse were consulted, the advice took on a more urgent tone. Table 2 provides examples of these forms of advice.

**Table 2:** Colorectal cancer advice typologies

Forms of family advice	Examples
Casual	[Did your husband advocate seeking more information or further testing?] Patient: “Well he figured I should go to the doctor more often.”
Urgent	Patient: “They all thought maybe it could’ve been a change of life, and I told them about my ulcers and they thought maybe that [could] be it…[W]hen the symptoms were getting worse and I wasn’t feeling any better, they said ‘*You need to go to the doctor’s and you need to go as soon as possible.*’”
Advice based on others’ experiences	[Advice from an aunt] Patient: “I said I told her I know something’s going on. I said, ‘I’m glad that you stopped by because you and I have the same symptoms going on.’ I said, ‘let me know what your doctor that you went to says.’”

A qualitative analysis of the interview responses further revealed that 92% of patients discussed their symptoms with a family member or friend. More than half (52%) confided in someone within 28 days of recognizing their symptoms, and 51% consulted with two or more confidants. Patients most often shared their symptoms with their spouses (51.6%), whereas neighbors or friends (22.2%), children (21.8%), and coworkers (14.3%) were consulted less often.

Confidants responded to the sharing of symptoms by confirming the patient’s symptom appraisal (56%) and advocating for the patient to obtain more information or tests (42.5%). Analysis indicated that nearly 67% of patients took action and sought medical attention after sharing the information about their symptoms with confidants. Table 3 provides these results.

**Table 3:** Outcomes of sharing symptoms with confidants

Result	Percentage of patients
Patient took action after confiding in others	66.7%
Confidant confirmed symptom appraisal	56.0%
Patient thought something worse was wrong after talking with others	48.4%
Confidants advocated getting more information/tests	42.5%
Patient was influenced by the personal health experiences of others	26.9%

Patients tend to seek counsel and validation from friends and family regarding their symptoms. As our data suggest, patients, in an attempt to try to “make sense” of their symptoms, speak to confidants. These data illustrate the important role that family and friends may play in diagnosis-seeking behaviors and the potential influence they may have on treatment-related decisions as well.

### Choosing treatment and care

Although the formal health-care system is predicated on patients following a treatment plan that is based on the best scientific evidence available, illnesses that are not acute and rely on patient and family caregiver care-seeking behaviors in the outpatient setting are subject to many other influences. In our study of patients with lung cancer, we found that family caregivers played influential roles in the treatment and care plans that patients follow and the relationships between physicians and patients. For example, 17% of patients with lung cancer were persuaded by their families to change doctors while being treated for the disease, largely because they were unhappy with their physician’s communication style or the treatment options provided [19].

Patients were also highly influenced by the preferences of family caregivers, although the mechanism of influence was sometimes conflict. The most common areas of conflict were: issues concerning lifestyle, especially smoking (34.4%); sharing/withholding information (17.5%); and treatment decisions (26.2%). Of interest is that the family caregivers most frequently urged patients to try alternative therapies or to take more (or less) aggressive treatments than recommended by their physicians [19]. For example, family caregivers often advocate the use of vitamins or other supplements; however, this can be a source of conflict with physicians who often do not hold the same beliefs about the use of supplements and may use disconfirming messaging (especially in the form of disparagement) to discourage patients from using them. However, there is evidence that this approach may serve to discourage patients from sharing information with their physicians; studies indicate that fewer than half of patients with cancer actually tell their physicians they are using some form of complementary or alternative medicine [20]. Among the lung cancer sample, one patient’s wife shared her experience in attempting to discuss complementary and alternative medicine with her husband’s health-care team:

“I had a question about why couldn’t he [the patient] take vitamins, and I didn’t ask the doctor and I should have…I said, [to the nursing team], ‘Well I want to get him on some vitamins,’ and they said, ‘I’d be careful of those vitamins,’ and so after we left I thought, ‘Why? Why can’t I give him some of the selenium and all that kind of stuff that I read is supposed to be good?’”

Another example is a spouse who was extremely involved in her husband’s treatment. Note that in the quote below she refers to “us” as she discussed her husband’s chemotherapy treatment.

“I had, prior to us getting into chemotherapy, I had gone to the health store and he was taking a combination of herbs and things that we had read about that might help, and I had talked to the person that runs the store, who I’ve talked to in the past about other problems, and she was telling me about a lot of people that use these…she was telling me about these things and Internet things, stuff you get, a combination of herbs you get from Canada that worked on this guy who was at [the] Clinic, and she said, ‘You can visit these Websites.’ She gave us Websites and stuff, so we had started him on some things just prior to the chemo.”

Family members also can become intrusive during the clinical encounter and interfere with the patient-physician relationship. Patients may not feel as though they can have candid conversations with their physicians, especially if their values or desired outcomes differ from those of their family members. Furthermore, patients may feel pressured by family members to select a particular treatment over another because it may be less burdensome to or disruptive for the family, despite the evidence that the other treatment is clinically superior. For example, some patients with cancer may reject radiation therapy because of the time involved and the number of outpatient visits. They may select a more aggressive treatment to prolong life because that is what their family members desire or expect. The quote below illustrates this point; the patient’s husband is adamant about attending the appointment and pursuing the most aggressive treatment, whereas it seems that the patient may not agree or has not made a similar determination at that point for herself:

“My husband insists on being there and I keep telling him it’s really not necessary, and he thinks 4 years are better than 2, and I guess he’s right, and he probably is figuring I wouldn’t tell him something, which might be true, but it would give me a time where I could talk to the doctor more frankly.”

## Conclusions

Illness, including its diagnosis and treatment, is not just a biological but also a social process. EBM needs to account for this duality and expand to incorporate sociocultural influences, such as family influences and patient values and preferences. Our research and the reported results of other studies [21-27] demonstrate that family members attend clinical encounters, offer opinions, and participate in treatment-related decision making. Thus, clinicians must consider the role family members play and the influence they may have on the patient’s treatment choices. Recognition of family influences may lead to more efficient and effective care. For example, family members may be a greater influence on how a patient presents his/her illness to a health-care provider or the kinds of treatment he/she seeks or accepts other than any supported by evidence-based research. Understanding these influences can help clinicians apply EBM as fully and as appropriately as possible.

As clinicians move to adopt evidence-based approaches to treatment, we cannot lose sight that any “disease,” although contained within an objective physical state, is experienced by patients as an “illness” in which their social and psychological contexts are critically important. To avoid a return to biological reductionism, I recommend that clinicians take the following steps as they evaluate and work with patients (and their families) to establish treatment plans:

**•** Assess patient and family explanatory models of symptoms and illness. Ask patients what they think may be causing their symptoms/illness, and listen to their explanations.

**•** Validate patient and family concerns. Reflect back what you have heard using statements such as, “I understand that you are concerned about…” or “I can see why that would be a concern.” Try to maintain eye contact, and express empathy.

**•** Iteratively check understanding by asking patients to explain or “teach back” to you, in their own words, what you have just explained [28]. Tell patients that you want to be sure you are covering everything they need to know so your questions do not sound like a “test” to the patient. This will also allow you to correct any misinformation in a nonjudgmental way. Doing this throughout the visit, and not just at the end, will help break information into more manageable “chunks” and make it less overwhelming.

**•** Use relational communication strategies to build rapport and shared meaning. While words are used to convey information, how they are used along with their corresponding nonverbal cues also communicates significant meaning [29]. Consider not only what patients are saying but also what they may not be saying and always confirm to avoid misunderstanding and conflict. Be cognizant of nonverbal cues and the messages being sent as a result.

**•** Discuss sources of information. Encourage patients to talk about what they have learned or know about their illness and about their source(s) for that information. Encouraging patients to share can allow you to reinforce pertinent information while dispelling any inaccurate or incorrect information. It is critically important that misinformation is addressed in a nonjudgmental way so that the conversation continues and patients do not feel belittled. Consider using this part of the discussion or interview to direct patients to reliable information sources [30]. Acknowledge and legitimate that patients may continue to function under a different explanatory paradigm. Understanding and accepting this difference allows you to work as effectively as possible with the patient.

**•** Consider how to incorporate patient values and needs in treatment plans. Ask patients what they hope to accomplish with treatment and what preferences or suggestions for treatment they may have [31]. Engage the patient in a discussion of the pros and cons of treatment(s) and have them relate them to their values and needs as appropriate and participate in shared decision making.

Communicating with patients in ways that elicit more than a description of physical symptoms has the potential to provide greater insight into the values, beliefs, and explanations that each patient brings to the clinical encounter. When combining this patient-centered approach with EBM, physicians can merge these paradigms and deliver care that is evidenced based while incorporating the values and preferences of patients and their families.

## List of abbreviations used

CRC: colorectal cancer; EBM: evidence-based medicine

## Competing interests

The author does not have any actual or potential conflict of interest including any financial, personal, or other relationships with other people or organizations within 3 years of beginning the submitted work that could inappropriately influence, or be perceived to influence, this manuscript.

## Declarations and disclaimer

The Eisenberg Conference Series 2012, *Supporting Informed Decision Making When Clinical Evidence and Conventional Wisdom Collide*, was conducted in Rockville, Maryland, by the John M. Eisenberg Center for Clinical Decisions and Communications Science, Baylor College of Medicine, Houston, Texas, for the Agency for Healthcare Research and Quality under Contract No. HHSA 290-2008-10015-C. Publication costs for this supplement were funded by this contract. The author of this article is responsible for its content. No statement may be construed as the official position of the Agency for Healthcare Research and Quality and of the U.S. Department of Health and Human Services.

This article has been published as part of *BMC Medical Informatics and Decision Making* Volume 13 Supplement 3, 2013: Articles from the Eisenberg Center Conference Series 2012: Supporting informed decision making when clinical evidence and conventional wisdom collide. The full contents of the supplement are available online at http://www.biomedcentral.com/bmcmedinformdecismak/supplements/13/S3.

## References

[B1] Sackett DL Rosenberg WM Gray JA Haynes RB Richardson WS Evidence based medicine: what it is and what it isn't BMJ 1996 13 71 72 10.1136/bmj.312.7023.71 8555924 PMC2349778

[B2] Gill VT Patient "demand" for medical interventions: exerting pressure for an offer in a primary care clinic visit Res Lang Soc Interact 2005 13 451 479 10.1207/s15327973rlsi3804_3

[B3] Epstein RM The science of patient-centered care J Fam Pract 2000 13 805 807 11032204

[B4] Robinson JH Callister LC Berry JA Dearing KA Patient-centered care and adherence: definitions and applications to improve outcomes J Am Acad Nurse Pract 2008 13 600 607 10.1111/j.1745-7599.2008.00360.x 19120591

[B5] Agency for Healthcare Research and Quality Patient centeredness National Healthcare Quality Report 2005 Rockville, MD: Agency for Healthcare Research and Quality http://archive.ahrq.gov/qual/nhqr05/fullreport/PatCen.htm

[B6] Anderson E Patient-centeredness: a new approach Nephr News Issues 2002 13 80 82 12452113

[B7] Beck RS Daughtridge R Sloane PD Physician-patient communication in the primary care office: a systematic review J Am Board Fam Pract 2002 13 25 38 11841136

[B8] Stewart M Brown JB Donner A McWhinney IR Oates J Weston WW Jordan J The impact of patient-centered care on outcomes Fam Pract 2000 13 796 804 11032203

[B9] Bensing J Bridging the gap: the separate worlds of evidence-based medicine and patient-centered medicine Patient Educ Couns 2000 13 17 25 10.1016/S0738-3991(99)00087-7 11013544

[B10] Kleinman A Eisenberg L Good B Culture, illness, and care: clinical lessons from anthropologic and cross-cultural research Ann Intern Med 1978 13 251 258 10.7326/0003-4819-88-2-251 626456

[B11] Loewe R Freeman J Interpreting diabetes mellitus: differences between patient and provider models of disease and their implications for clinical practice Cult Med Psychiatry 2000 13 379 401 10.1023/A:1005611207687 11128624

[B12] Torgerson DJ Sibbald B Understanding controlled trials: what is a patient preference trial BMJ 1998 13 360 10.1136/bmj.316.7128.360 9487173 PMC2665528

[B13] Sayers SL White T Zubritsky C Oslin DW Family involvement in the care of healthy medical outpatients Fam Pract 2006 13 317 324 10.1093/fampra/cmi114 16461451

[B14] Siminoff LA Zyzanski SJ Rose JH Zhang AY The Cancer Communication Assessment Tool for Patients and Families (CCAT-PF): a new measure Psychooncology 2008 13 1216 1224 10.1002/pon.1350 18504807 PMC2830149

[B15] Lewis N Gray SW Freres DR Hornik RC Examining cross-source engagement with cancer-related information and its impact on doctor-patient relations Health Commun 2009 13 723 734 10.1080/10410230903264030 20183381 PMC2950967

[B16] Siminoff LA Rogers HL Thomson MD Dumenci L Harris-Haywood S Doctor, what's wrong with me? Factors that delay the diagnosis of colorectal cancer Patient Educ Couns 2011 13 352 358 10.1016/j.pec.2011.05.002 21621950 PMC3159771

[B17] Zhang AY Zyzanski SJ Siminoff LA Differential patient-caregiver opinions of treatment and care for advanced lung cancer patients Soc Sci Med 2010 13 1155 1158 10.1016/j.socscimed.2009.12.023 20137849 PMC2840198

[B18] Thomson MD Siminoff LA Longo DR Internet use for prediagnosis symptom appraisal by colorectal cancer patients Health Educ Behav 2012 13 583 588 10.1177/1090198111423941 21990571 PMC3521844

[B19] Zhang AY Siminoff LA The role of the family in treatment decision making by patients with cancer Onc Nurs Forum 2003 13 1022 1028 10.1188/03.ONF.1022-1028 14603359

[B20] Schofield PE Juraskova I Butow PN How oncologists discuss complementary therapy use with their patients: an audio-tape audit Support Care Cancer 2003 13 348 355 12712375 10.1007/s00520-002-0420-x

[B21] Basch EM Thaler HT Shi W Yakren S Schrag D Use of information resources by patients with cancer and their companions Cancer 2004 13 2476 2483 10.1002/cncr.20261 15160355

[B22] Botelho RJ Lue BH Fiscella K Family involvement in routine health care: a survey of patients' behaviors and preferences J Fam Pract 1996 13 572 576 8656167

[B23] Brown JB Brett P Stewart M Marshall JN Roles and influence of people who accompany patients on visits to the doctor Can Fam Physician 1998 13 1644 1650 9721420 PMC2277722

[B24] Clayman ML Roter D Wissow LS Bandeen-Roche K Autonomy-related behaviors of patient companions and their effect on decision-making activity in geriatric primary care visits Soc Sci Med 2005 13 1583 1591 10.1016/j.socscimed.2004.08.004 15652689

[B25] Ho A Relational autonomy or undue pressure? Family’s role in medical decision-making Scand J Caring Sci 2008 13 128 135 10.1111/j.1471-6712.2007.00561.x 18269432

[B26] Rosland AM Piette JD Choi H Heisler M Family and friend participation in primary care visits of patients with diabetes or heart failure: patient and physician determinants and experiences Med Care 2011 13 37 45 10.1097/MLR.0b013e3181f37d28 21102357 PMC3712763

[B27] Schilling LM Scatena L Steiner JF Albertson GA Lin CT Cyran L Ware L Anderson RJ The third person in the room: frequency, role, and influence of companions during primary care medical encounters J Fam Pract 2002 13 685 690 12184964

[B28] Weiss B Health Literacy and Patient Safety: Help Patients Understand—Manual for Clinicians 2007 Chicago: American Medical Association Foundation

[B29] Ong LM De Haes JC Hoos AM Lammes FB Doctor-patient communication: a review of the literature Soc Sci Med 1995 13 903 918 10.1016/0277-9536(94)00155-M 7792630

[B30] Wald HS Dube CE Anthony DC Untangling the Web—the impact of Internet use on health care and the physician-patient relationship Patient Educ Couns 2007 13 218 224 10.1016/j.pec.2007.05.016 17920226

[B31] Benbassat J Baumal R What is empathy, and how can it be promoted during clinical clerkships? Acad Med 2004 13 832 839 10.1097/00001888-200409000-00004 15326005

